# Supplementary material for: Building the vertebrate codex using the gene breaking protein trap library
Source: eLife. 2020 Aug 11;9:e54572. doi: 10.7554/eLife.54572 (PMC7486118; doi:10.7554/eLife.54572)
Supplement: Supplementary file 6. — Individual worksheets in Figure 4—source data 1 and Figure 4—figure supplement 1—source data 1 represent the individual ‘.csv’ files read into R to perform these analyses and are named accordingly. [file elife-54572-supp6.pdf]

Supplementary File 6 for Building the Vertebrate Codex using the Gene Breaking Protein Trap Library

Ichino et al.

1/23/2020

Calcium Imaging Data Analyses

Figure 4K—Peak by fish

```
#Reading in fish averaged data
fish348 <- read.csv("~/Rdata/fish348.csv")
fish348$fish <- as.factor(fish348$fish)
summary(fish348)
```

```
##       fish          gene         peak              num            totcell
##  1      : 1   gbt348hom:16   Min.   :0.0000   Min.   :1.000   Min.   :1.000
##  2      : 1   wt       :19   1st Qu.:0.0596   1st Qu.:1.000   1st Qu.:2.000
##  3      : 1                  Median :0.7818   Median :1.000   Median :2.000
##  4      : 1                  Mean   :1.9665   Mean   :2.171   Mean   :3.229
##  5      : 1                  3rd Qu.:3.8626   3rd Qu.:3.000   3rd Qu.:5.000
##  6      : 1                  Max.   :8.2873   Max.   :9.000   Max.   :8.000
##  (Other):29
```

```
#Mann-Whitney U-test
Utest_fig4k <- wilcox.test(peak~gene, data = fish348, alternative = "two.sided", paired= FALSE)
```

```
## Warning in wilcox.test.default(x = c(0.43687541, 0.028478701, 0.004881698, :
## cannot compute exact p-value with ties
```

```
Utest_fig4k
```

```
##
##  Wilcoxon rank sum test with continuity correction
##
## data:  peak by gene
## W = 6, p-value = 1.448e-06
## alternative hypothesis: true location shift is not equal to 0
```

```
#Asymptotic Mann-Whitney U-test due to existence of ties
```

```
library(coin)
Utest2_fig4k <- wilcox_test(peak~gene, data = fish348)
Utest2_fig4k
```

```
##
##  Asymptotic Wilcoxon-Mann-Whitney Test
##
## data:  peak by gene (gbt348hom, wt)
## Z = -4.8349, p-value = 1.332e-06
```

```
## alternative hypothesis: true mu is not equal to 0
```

```r
#Analysis of effect size with Cohen's d
library(effsize)
cohdfig4k<- cohen.d(peak~gene, data = fish348)
cohdfig4k
```

```
##
## Cohen's d
##
## d estimate: -1.829053 (large)
## 95 percent confidence interval:
##     lower     upper
## -2.650261 -1.007846
```

```r
#Because "gbt348hom" comes before "wt" in the alphabet R computes a "negative" Cohen's d in this case.
```

```r
library(outliers)
fish348wt <- read.csv("~/Rdata/fish348WT.csv")
fish348gbt <- read.csv("~/Rdata/fish348GBT.csv")
fish348wt$fish <- as.factor(fish348wt$fish)
fish348gbt$fish <- as.factor(fish348gbt$fish)
summary(fish348wt)
```

```
##       fish    gene         peak             num           totcell
##  1      : 1   wt:19   Min.   :0.1664   Min.   :1.000   Min.   :1.000
##  2      : 1           1st Qu.:1.1241   1st Qu.:1.000   1st Qu.:2.000
##  3      : 1           Median :3.6167   Median :2.000   Median :3.000
##  4      : 1           Mean   :3.4900   Mean   :2.158   Mean   :3.421
##  5      : 1           3rd Qu.:5.0210   3rd Qu.:2.500   3rd Qu.:5.000
##  6      : 1           Max.   :8.2873   Max.   :9.000   Max.   :7.000
##  (Other):13
```

```r
summary(fish348gbt)
```

```
##       fish           gene         peak              num           totcell
##  20     : 1   gbt348hom:16   Min.   :0.00000   Min.   :1.000   Min.   :1.00
##  21     : 1                  1st Qu.:0.01816   1st Qu.:1.000   1st Qu.:1.75
##  22     : 1                  Median :0.04961   Median :1.000   Median :2.00
##  23     : 1                  Mean   :0.15745   Mean   :2.188   Mean   :3.00
##  24     : 1                  3rd Qu.:0.26605   3rd Qu.:3.250   3rd Qu.:4.25
##  25     : 1                  Max.   :0.78182   Max.   :8.000   Max.   :8.00
##  (Other):10
```

```r
#testing for an outlier in the WT dataset
grubbs.test(fish348wt$peak, type = 10)
```

```
##
##  Grubbs test for one outlier
##
## data:  fish348wt$peak
## G = 1.95118, U = 0.77674, p-value = 0.3901
## alternative hypothesis: highest value 8.287278317 is an outlier
```

```r
#testing for an outlier in the GBT348Hom dataset
grubbs.test(fish348gbt$peak, type = 10)
```

```
##
```

```
##  Grubbs test for one outlier
##
## data:  fish348gbt$peak
## G = 2.81593, U = 0.43613, p-value = 0.006409
## alternative hypothesis: highest value 0.781822916 is an outlier
```

```
#Asymptotic Mann-Whitney U test excluding outlier in GBT348Hom dataset
fish348x <- read.csv("~/Rdata/fish348x.csv")
fish348x$fish <- as.factor(fish348x$fish)
summary(fish348x)
```

```
##       fish            gene          peak               num            totcell
##  1      : 1   gbt348hom:15   Min.   :0.00000   Min.   :1.000   Min.   :1.000
##  2      : 1   wt       :19   1st Qu.:0.05732   1st Qu.:1.000   1st Qu.:2.000
##  3      : 1                  Median :0.77782   Median :1.000   Median :2.000
##  4      : 1                  Mean   :2.00137   Mean   :2.147   Mean   :3.235
##  5      : 1                  3rd Qu.:3.98558   3rd Qu.:2.750   3rd Qu.:5.000
##  6      : 1                  Max.   :8.28728   Max.   :9.000   Max.   :8.000
##  (Other):28
```

```
Utest3_fig4k <- wilcox_test(peak~gene, data = fish348x)
Utest3_fig4k
```

```
##
##  Asymptotic Wilcoxon-Mann-Whitney Test
##
## data:  peak by gene (gbt348hom, wt)
## Z = -4.8042, p-value = 1.554e-06
## alternative hypothesis: true mu is not equal to 0
```

Figure 4L—Peak-width at half max

```
#Reading in individual kinetic data
kin348 <- read.csv("~/Rdata/348kin.csv")
kin348$cell <- as.factor(kin348$cell)
summary(kin348)
```

```
##       cell            gene          peak               width
##  1      : 1   gbt348hom:16   Min.   : 0.00918   Min.   : 1.098
##  2      : 1   wt       :32   1st Qu.: 0.25633   1st Qu.: 3.080
##  3      : 1                  Median : 2.81048   Median : 4.249
##  4      : 1                  Mean   : 3.42464   Mean   : 5.477
##  5      : 1                  3rd Qu.: 5.71602   3rd Qu.: 6.821
##  6      : 1                  Max.   :12.25581   Max.   :22.600
##  (Other):42
##       rise              decay
##  Min.   : 0.3989   Min.   :0.488
##  1st Qu.: 0.6200   1st Qu.:1.308
##  Median : 0.7465   Median :2.085
##  Mean   : 1.7208   Mean   :2.590
##  3rd Qu.: 1.5234   3rd Qu.:3.686
##  Max.   :22.9649   Max.   :8.422
##                    NA's   :1
```

```
#Running Mann-Whitney U-test
Utest_fig4l <- wilcox.test(width~gene, data = kin348, alternative = "two.sided", paired= FALSE)
```

```
Utest_fig4l
```

```
##
##  Wilcoxon rank sum test
##
## data:  width by gene
## W = 299, p-value = 0.3565
## alternative hypothesis: true location shift is not equal to 0
```

```
kin348wt <- read.csv("~/Rdata/348kinWT.csv")
kin348gbt <- read.csv("~/Rdata/348kinGBT.csv")
kin348wt$cell <- as.factor(kin348wt$cell)
kin348gbt$cell <- as.factor(kin348gbt$cell)
summary(kin348wt)
```

```
##       cell    gene         peak             width             rise
##  1      : 1   wt:32   Min.   : 0.2187   Min.   : 1.098   Min.   :0.3989
##  2      : 1           1st Qu.: 2.9942   1st Qu.: 2.923   1st Qu.:0.6058
##  3      : 1           Median : 4.6847   Median : 3.981   Median :0.6780
##  4      : 1           Mean   : 4.9927   Mean   : 4.944   Mean   :0.8299
##  5      : 1           3rd Qu.: 7.2304   3rd Qu.: 6.821   3rd Qu.:0.7370
##  6      : 1           Max.   :12.2558   Max.   :16.586   Max.   :3.7309
##  (Other):26
##      decay
##  Min.   :0.488
##  1st Qu.:1.479
##  Median :2.125
##  Mean   :2.639
##  3rd Qu.:3.786
##  Max.   :7.485
##
```

```
summary(kin348gbt)
```

```
##       cell           gene         peak              width
##  33     : 1   gbt348hom:16   Min.   :0.00918   Min.   : 1.323
##  34     : 1                  1st Qu.:0.05658   1st Qu.: 3.667
##  35     : 1                  Median :0.09320   Median : 4.539
##  36     : 1                  Mean   :0.28860   Mean   : 6.545
##  37     : 1                  3rd Qu.:0.30463   3rd Qu.: 6.199
##  38     : 1                  Max.   :1.81724   Max.   :22.600
##  (Other):10
##       rise              decay
##  Min.   : 0.7755   Min.   :0.5715
##  1st Qu.: 1.4729   1st Qu.:1.1451
##  Median : 1.5578   Median :1.9446
##  Mean   : 3.5027   Mean   :2.4849
##  3rd Qu.: 3.5286   3rd Qu.:2.4320
##  Max.   :22.9649   Max.   :8.4222
##                    NA's   :1
```

```
#testing for an outlier in the WT dataset
grubbs.test(kin348wt$width, type = 10)
```

```
##
##  Grubbs test for one outlier
```

```
##
## data:  kin348wt$width
## G = 3.55964, U = 0.57807, p-value = 0.0009204
## alternative hypothesis: highest value 16.58582 is an outlier
```

```
#testing for an outlier in the GBT348Hom dataset
grubbs.test(kin348gbt$width, type = 10)
```

```
##
##  Grubbs test for one outlier
##
## data:  kin348gbt$width
## G = 2.79613, U = 0.44403, p-value = 0.00731
## alternative hypothesis: highest value 22.6 is an outlier
```

```
#Mann-Whitney U test excluding outlier in both datasets
kin348x <- read.csv("~/Rdata/348kinx.csv")
kin348x$cell <- as.factor(kin348x$cell)
summary(kin348x)
```

```
##       cell           gene         peak              width
##  1      : 1   gbt348hom:15   Min.   : 0.00918   Min.   : 1.098
##  2      : 1   wt       :31   1st Qu.: 0.30463   1st Qu.: 3.040
##  3      : 1                  Median : 2.81048   Median : 4.178
##  4      : 1                  Mean   : 3.47478   Mean   : 4.864
##  5      : 1                  3rd Qu.: 6.00780   3rd Qu.: 6.263
##  6      : 1                  Max.   :12.25581   Max.   :17.962
##  (Other):40
##       rise             decay
##  Min.   : 0.3989   Min.   :0.488
##  1st Qu.: 0.6153   1st Qu.:1.288
##  Median : 0.7227   Median :2.058
##  Mean   : 1.6058   Mean   :2.526
##  3rd Qu.: 1.4729   3rd Qu.:3.604
##  Max.   :22.9649   Max.   :8.422
##
```

```
Utest2_fig4l <- wilcox.test(width~gene, data = kin348x)
Utest2_fig4l
```

```
##
##  Wilcoxon rank sum test
##
## data:  width by gene
## W = 266, p-value = 0.4437
## alternative hypothesis: true location shift is not equal to 0
```

Figure 4M—Rise time

```
#Running Mann-Whitney U-test
Utest_fig4m <- wilcox.test(rise~gene, data = kin348, alternative = "two.sided", paired= FALSE)
Utest_fig4m
```

```
##
##  Wilcoxon rank sum test
##
```

```
## data:  rise by gene
## W = 482, p-value = 2.427e-08
## alternative hypothesis: true location shift is not equal to 0
```

```
#Analysis of effect size with Cohen's d
cohdfig4m<- cohen.d(rise~gene, data = kin348)
cohdfig4m
```

```
##
## Cohen's d
##
## d estimate: 0.8663981 (large)
## 95 percent confidence interval:
##     lower     upper
## 0.2248897 1.5079064
```

```
#testing for an outlier in the WT dataset
grubbs.test(kin348wt$rise, type = 10)
```

```
##
##  Grubbs test for one outlier
##
## data:  kin348wt$rise
## G = 4.58245, U = 0.30077, p-value = 4.068e-08
## alternative hypothesis: highest value 3.730921631 is an outlier
```

```
#testing for an outlier in the GBT348Hom dataset
grubbs.test(kin348gbt$rise, type = 10)
```

```
##
##  Grubbs test for one outlier
##
## data:  kin348gbt$rise
## G = 3.654834, U = 0.050111, p-value = 1.36e-09
## alternative hypothesis: highest value 22.9649 is an outlier
```

```
#Mann-Whitney U test excluding outlier in both datasets
kin348xr <- read.csv("~/Rdata/348kinxr.csv")
kin348xr$cell <- as.factor(kin348xr$cell)
summary(kin348xr)
```

```
##       cell            gene         peak               width
##  1      : 1   gbt348hom:15   Min.   : 0.01099   Min.   : 1.098
##  2      : 1   wt       :31   1st Qu.: 0.30463   1st Qu.: 3.040
##  3      : 1                  Median : 2.81048   Median : 4.178
##  4      : 1                  Mean   : 3.47572   Mean   : 4.964
##  5      : 1                  3rd Qu.: 6.00780   3rd Qu.: 6.263
##  6      : 1                  Max.   :12.25581   Max.   :22.600
##  (Other):40
##       rise            decay
##  Min.   :0.3989   Min.   :0.488
##  1st Qu.:0.6153   1st Qu.:1.268
##  Median :0.7227   Median :2.032
##  Mean   :1.2153   Mean   :2.395
##  3rd Qu.:1.4729   3rd Qu.:3.438
##  Max.   :5.0019   Max.   :7.485
##                   NA's   :1
```

```r
Utest2_fig4m <- wilcox.test(rise~gene, data = kin348xr)
Utest2_fig4m
```

```
##
##  Wilcoxon rank sum test
##
## data:  rise by gene
## W = 449, p-value = 3.572e-09
## alternative hypothesis: true location shift is not equal to 0
```

Figure 4N—Decay time

```r
#Running Mann-Whitney U-test
Utest_fig4n <- wilcox.test(decay~gene, data = kin348, alternative = "two.sided", paired= FALSE)
Utest_fig4n
```

```
##
##  Wilcoxon rank sum test
##
## data:  decay by gene
## W = 217, p-value = 0.6114
## alternative hypothesis: true location shift is not equal to 0
```

```r
#testing for an outlier in the WT dataset
grubbs.test(kin348wt$decay, type = 10)
```

```
##
##  Grubbs test for one outlier
##
## data:  kin348wt$decay
## G = 2.83123, U = 0.73308, p-value = 0.03947
## alternative hypothesis: highest value 7.48504248 is an outlier
```

```r
#testing for an outlier in the GBT348Hom dataset
grubbs.test(kin348gbt$decay, type = 10)
```

```
##
##  Grubbs test for one outlier
##
## data:  kin348gbt$decay
## G = 2.80380, U = 0.39837, p-value = 0.005085
## alternative hypothesis: highest value 8.422230469 is an outlier
```

```r
#Mann-Whitney U test excluding outlier in both datasets
kin348xd <- read.csv("~/Rdata/348kinxd.csv")
kin348xd$cell <- as.factor(kin348xd$cell)
summary(kin348xd)
```

```
##       cell            gene          peak               width
##  1      : 1   gbt348hom:15   Min.   : 0.01099   Min.   : 1.098
##  2      : 1   wt       :31   1st Qu.: 0.30463   1st Qu.: 3.040
##  3      : 1                  Median : 2.81048   Median : 4.178
##  4      : 1                  Mean   : 3.47141   Mean   : 5.099
##  5      : 1                  3rd Qu.: 6.00780   3rd Qu.: 6.263
##  6      : 1                  Max.   :12.25581   Max.   :22.600
##  (Other):40
```

```
##       rise            decay
##  Min.   :0.3989   Min.   :0.488
##  1st Qu.:0.6153   1st Qu.:1.268
##  Median :0.7227   Median :2.032
##  Mean   :1.2789   Mean   :2.351
##  3rd Qu.:1.5124   3rd Qu.:3.438
##  Max.   :5.0019   Max.   :5.541
##                   NA's   :1
```

```
Utest2_fig4n <- wilcox.test(decay~gene, data = kin348xd)
Utest2_fig4n
```

```
##
##  Wilcoxon rank sum test
##
## data:  decay by gene
## W = 185, p-value = 0.4445
## alternative hypothesis: true location shift is not equal to 0
```

Figure 4—figure supplement 1A—Peak by cell

```
#Reading in cell averaged data
cell348 <- read.csv("~/Rdata/cell348.csv")
cell348$cell <- as.factor(cell348$cell)
summary(cell348)
```

```
##       cell            gene           peak              peaknum
##  1       :  1   gbt348hom:48   Min.   :0.00000   Min.   :0.000
##  2       :  1   wt       :64   1st Qu.:0.03449   1st Qu.:0.000
##  3       :  1                  Median :0.59974   Median :1.000
##  4       :  1                  Mean   :1.71208   Mean   :1.643
##  5       :  1                  3rd Qu.:2.50715   3rd Qu.:2.250
##  6       :  1                  Max.   :8.42028   Max.   :9.000
##  (Other):106
```

```
#Running Mann-Whitney U-test
Utest_fig4s1a <- wilcox.test(peak~gene, data = cell348, alternative = "two.sided", paired= FALSE)
Utest_fig4s1a
```

```
##
##  Wilcoxon rank sum test with continuity correction
##
## data:  peak by gene
## W = 90, p-value < 2.2e-16
## alternative hypothesis: true location shift is not equal to 0
```

```
#Analysis of effect size with Cohen's d
cohdfig4s1a<- cohen.d(peak~gene, data = cell348)
cohdfig4s1a
```

```
##
## Cohen's d
##
## d estimate: -1.445318 (large)
## 95 percent confidence interval:
##     lower     upper
```

```
## -1.869360 -1.021276
```

```r
#Because "gbt348hom" comes before "wt" in the alphabet R computes a "negative" Cohen's d in this case.
cell348wt <- read.csv("~/Rdata/cell348WT.csv")
cell348gbt <- read.csv("~/Rdata/cell348GBT.csv")
cell348wt$cell <- as.factor(cell348wt$cell)
cell348gbt$cell <- as.factor(cell348gbt$cell)
summary(cell348wt)
```

```
##       cell     gene         peak             peaknum
##  1       : 1   wt:64   Min.   :0.06406   Min.   :1.00
##  2       : 1           1st Qu.:0.78922   1st Qu.:1.00
##  3       : 1           Median :1.98992   Median :2.00
##  4       : 1           Mean   :2.90744   Mean   :2.25
##  5       : 1           3rd Qu.:4.55220   3rd Qu.:3.00
##  6       : 1           Max.   :8.42028   Max.   :9.00
##  (Other):58
```

```r
summary(cell348gbt)
```

```
##       cell            gene         peak              peaknum
##  65      : 1   gbt348hom:48   Min.   :0.000000   Min.   :0.0000
##  66      : 1                  1st Qu.:0.003265   1st Qu.:0.0000
##  67      : 1                  Median :0.016491   Median :0.0000
##  68      : 1                  Mean   :0.118277   Mean   :0.8333
##  69      : 1                  3rd Qu.:0.061702   3rd Qu.:1.0000
##  70      : 1                  Max.   :1.898103   Max.   :8.0000
##  (Other):42
```

```r
#testing for an outlier in the WT dataset
grubbs.test(cell348wt$peak, type = 10)
```

```
##
##  Grubbs test for one outlier
##
## data:  cell348wt$peak
## G = 2.17353, U = 0.92382, p-value = 0.8727
## alternative hypothesis: highest value 8.42028226 is an outlier
```

```r
#testing for an outlier in the GBT348Hom dataset
grubbs.test(cell348gbt$peak, type = 10)
```

```
##
##  Grubbs test for one outlier
##
## data:  cell348gbt$peak
## G = 5.83977, U = 0.25897, p-value = 1.066e-13
## alternative hypothesis: highest value 1.898102954 is an outlier
```

```r
#Mann-Whitney U test excluding outlier in GBT348Hom dataset
cell348x <- read.csv("~/Rdata/cell348x.csv")
cell348x$cell <- as.factor(cell348x$cell)
summary(cell348x)
```

```
##       cell             gene          peak              peaknum
##  1       :  1   gbt348hom:47   Min.   :0.00000   Min.   :0.000
##  2       :  1   wt       :64   1st Qu.:0.03404   1st Qu.:0.000
##  3       :  1                  Median :0.51984   Median :1.000
```

```
##  4      :  1                 Mean   :1.71041   Mean   :1.631
##  5      :  1                 3rd Qu.:2.53931   3rd Qu.:2.000
##  6      :  1                 Max.   :8.42028   Max.   :9.000
##  (Other):105
```

```
Utest2_fig4s1a <- wilcox.test(peak~gene, data = cell348x)
Utest2_fig4s1a
```

```
##
##  Wilcoxon rank sum test with continuity correction
##
## data:  peak by gene
## W = 58, p-value < 2.2e-16
## alternative hypothesis: true location shift is not equal to 0
```

Figure 4—figure supplement 1B—Response number by cell

```
#Running Mann-Whitney U-test
Utest_fig4s1b <- wilcox.test(peaknum~gene, data = cell348, alternative = "two.sided", paired= FALSE)
Utest_fig4s1b
```

```
##
##  Wilcoxon rank sum test with continuity correction
##
## data:  peaknum by gene
## W = 518.5, p-value = 7.861e-10
## alternative hypothesis: true location shift is not equal to 0
```

```
#Analysis of effect size with Cohen's d
cohdfig4s1b<- cohen.d(peaknum~gene, data = cell348)
cohdfig4s1b
```

```
##
## Cohen's d
##
## d estimate: -0.9310606 (large)
## 95 percent confidence interval:
##      lower      upper
## -1.3290369 -0.5330843
```

```
#Because "gbt348hom" comes before "wt" in the alphabet R computes a "negative" Cohen's d in this case.
#testing for an outlier in the WT dataset
grubbs.test(cell348wt$peaknum, type = 10)
```

```
##
##  Grubbs test for one outlier
##
## data:  cell348wt$peaknum
## G = 4.49604, U = 0.67404, p-value = 2.684e-05
## alternative hypothesis: highest value 9 is an outlier
```

```
#testing for an outlier in the GBT348Hom dataset
grubbs.test(cell348gbt$peaknum, type = 10)
```

```
##
##  Grubbs test for one outlier
##
```

```
## data:  cell348gbt$peaknum
## G = 4.62880, U = 0.53443, p-value = 2.219e-06
## alternative hypothesis: highest value 8 is an outlier
```

```
#Mann-Whitney U test excluding outlier in both datasets
cell348xp <- read.csv("~/Rdata/cell348xp.csv")
cell348xp$cell <- as.factor(cell348xp$cell)
summary(cell348xp)
```

```
##       cell           gene          peak            peaknum
##  1      :  1   gbt348hom:47   Min.   :0.0000   Min.   :0.000
##  2      :  1   wt       :63   1st Qu.:0.0336   1st Qu.:0.000
##  3      :  1                  Median :0.5771   Median :1.000
##  4      :  1                  Mean   :1.7226   Mean   :1.518
##  5      :  1                  3rd Qu.:2.5715   3rd Qu.:2.000
##  6      :  1                  Max.   :8.4203   Max.   :9.000
##  (Other):104
```

```
Utest2_fig4s1b <- wilcox.test(peaknum~gene, data = cell348xp)
Utest2_fig4s1b
```

```
##
##  Wilcoxon rank sum test with continuity correction
##
## data:  peaknum by gene
## W = 456.5, p-value = 1.909e-10
## alternative hypothesis: true location shift is not equal to 0
```

Power Analyses For Calcium Imaging Experiments

```
#reading in peak dF/F0 by cell from first two trials
dataset1 <- read.csv("~/Rdata/dataset1xy.csv")
summary(dataset1)
```

```
##       data          genotype
##  Min.   :0.00000   Hom:27
##  1st Qu.:0.01282   WT :32
##  Median :0.24896
##  Mean   :0.77150
##  3rd Qu.:0.99591
##  Max.   :5.98587
```

```
#Two-sample t-test assuming equal variance to calculate the effect size using Cohen's d
result1 <- t.test(data~genotype, data = dataset1, var.equal = TRUE)
result1
```

```
##
##  Two Sample t-test
##
## data:  data by genotype
## t = -5.1022, df = 57, p-value = 4.008e-06
## alternative hypothesis: true difference in means is not equal to 0
## 95 percent confidence interval:
##  -1.8439141 -0.8044921
## sample estimates:
## mean in group Hom  mean in group WT
```

```
##        0.05328905        1.37749211
```

```r
#using Cohen's d to calculate the effect size. (This formula assumes equal variance)
abs(result1$statistic)*sqrt((32+27)/(32*27))
```

```
##        t
## 1.333297
```

```r
# load "pwr" package
library(pwr)
# calculate the power of this experiment based upon cells and effect size
pwr.t2n.test(n1=32,n2=27, d=1.333297, sig.level=0.05, power=NULL)
```

```
##
##      t test power calculation
##
##              n1 = 32
##              n2 = 27
##               d = 1.333297
##       sig.level = 0.05
##           power = 0.9988734
##     alternative = two.sided
```

```r
# use effect size to calculate the number of cells required for power = 0.8
pwr.t.test(n=NULL, d=1.333297, sig.level = 0.05, power = 0.8,type = "two.sample")
```

```
##
##      Two-sample t test power calculation
##
##               n = 9.889524
##               d = 1.333297
##       sig.level = 0.05
##           power = 0.8
##     alternative = two.sided
##
## NOTE: n is number in *each* group
```

```r
#calculating experimental delta off of the t.test
result1$estimate[2]-result1$estimate[1]
```

```
## mean in group WT
##         1.324203
```

```r
#calculating experimental standard deviation
sd(dataset1$data)
```

```
## [1] 1.188332
```

```r
# use observed difference and standard deviation to calculate the cells needed for power = 0.8
power.t.test(n=NULL, delta = 1.324203, sd=1.188332, sig.level = 0.05, power=0.8)
```

```
##
##      Two-sample t test power calculation
##
##               n = 13.67198
##           delta = 1.324203
##              sd = 1.188332
##       sig.level = 0.05
##           power = 0.8
```

```
##     alternative = two.sided
##
## NOTE: n is number in *each* group
```

```r
#reading in peak dF/F0 by fish from first two trials
dataset2 <-read.csv("~/Rdata/dataset2xy.csv")
summary(dataset2)
```

```
##       data          genotype
##  Min.   :0.004882   Hom:5
##  1st Qu.:0.036329   WT :6
##  Median :0.436875
##  Mean   :0.817645
##  3rd Qu.:1.512719
##  Max.   :2.353572
```

```r
#Two-sample t-test assuming equal variance to calculate the effect size using Cohen's d
result2 <- t.test(data~genotype, data = dataset2, var.equal=TRUE)
result2
```

```
##
##  Two Sample t-test
##
## data:  data by genotype
## t = -3.079, df = 9, p-value = 0.01316
## alternative hypothesis: true difference in means is not equal to 0
## 95 percent confidence interval:
##  -2.2596843 -0.3455981
## sample estimates:
## mean in group Hom  mean in group WT
##         0.1071131         1.4097544
```

```r
#using Cohen's d to calculate the effect size. (This formula assumes equal variance)
abs(result2$statistic)*sqrt((6+5)/(6*5))
```

```
##        t
## 1.864455
```

```r
#calculate the power of this experiment based upon fish and effect size
pwr.t2n.test(n1=6,n2=5, d=1.864455, sig.level=0.05, power=NULL)
```

```
##
##      t test power calculation
##
##              n1 = 6
##              n2 = 5
##               d = 1.864455
##       sig.level = 0.05
##           power = 0.7821757
##     alternative = two.sided
```

```r
# use effect size to calculate the number of fish required for power = 0.8
pwr.t.test(n=NULL, d=1.864455, sig.level = 0.05, power=0.8, type = "two.sample")
```

```
##
##      Two-sample t test power calculation
##
##               n = 5.658288
```

```
##                d = 1.864455
##        sig.level = 0.05
##            power = 0.8
##      alternative = two.sided
##
## NOTE: n is number in *each* group
```

```r
#calculating experimental delta off of the t.test
result2$estimate[2]-result2$estimate[1]
```

```
## mean in group WT
##         1.302641
```

```r
#calculating experimental standard deviation
sd(dataset2$data)
```

```
## [1] 0.9497955
```

```r
# use observed difference and standard deviation to calculate the fish needed for power = 0.8
power.t.test(n=NULL, delta = 1.302641, sd = 0.9497955, sig.level = 0.05, power=0.8)
```

```
##
##      Two-sample t test power calculation
##
##                n = 9.409926
##            delta = 1.302641
##               sd = 0.9497955
##        sig.level = 0.05
##            power = 0.8
##      alternative = two.sided
##
## NOTE: n is number in *each* group
```
