# Supplementary material for: Building the vertebrate codex using the gene breaking protein trap library
Source: eLife. 2020 Aug 11;9:e54572. doi: 10.7554/eLife.54572 (PMC7486118; doi:10.7554/eLife.54572)
Supplement: Transparent reporting form [file elife-54572-transrepform.docx]

***eLife’s* transparent reporting form**

We encourage authors to provide detailed information *within their submission* to facilitate the interpretation and replication of experiments. Authors can upload supporting documentation to indicate the use of appropriate reporting guidelines for health-related research (see [EQUATOR Network](http://www.equator-network.org/%20)), life science research (see the [BioSharing Information Resource](https://biosharing.org/)), or the [ARRIVE guidelines](http://www.plosbiology.org/article/info:doi/10.1371/journal.pbio.1000412) for reporting work involving animal research. Where applicable, authors should refer to any relevant reporting standards documents in this form.

If you have any questions, please consult our Journal Policies and/or contact us: [editorial@elifesciences.org](mailto:editorial@elifesciences.org).

**Sample-size estimation**

- You should state whether an appropriate sample size was computed when the study was being designed
- You should state the statistical method of sample size computation and any required assumptions
- If no explicit power analysis was used, you should describe how you decided what sample (replicate) size (number) to use

Please outline where this information can be found within the submission (e.g., sections or figure legends), or explain why this information doesn’t apply to your submission:

- Sample-size estimation procedures for calcium imaging studies can be found in the “Statistical Analysis” section of Materials and Methods and in Supplementary file 2 that contains both the R code and output for sample size estimation.
- No explicit power analysis was used to quantify knockdown efficiency of *lrpprc* mRNA in *lrpprc^mn0235Gt^*^/^*^mn0235Gt^* animals compared to WT animals. Sample size for this experiment was based on prior protocols for quantitative measurement of transcript levels in zebrafish larvae and mutants.

**Replicates**

- You should report how often each experiment was performed
- You should include a definition of biological versus technical replication
- The data obtained should be provided and sufficient information should be provided to indicate the number of independent biological and/or technical replicates
- If you encountered any outliers, you should describe how these were handled
- Criteria for exclusion/inclusion of data should be clearly stated
- High-throughput sequence data should be uploaded before submission, with a private link for reviewers provided (these are available from both GEO and ArrayExpress)

Please outline where this information can be found within the submission (e.g., sections or figure legends), or explain why this information doesn’t apply to your submission:

- Replicate information for calcium imaging experiments can be found in the “Calcium Imaging” section of Materials and Methods along with the figure legends for Figure 4 and Figure 4—Figure Supplement 1.
- Outlier analysis and treatment can be found in the “Statistical Analysis” section of Materials and Methods and the outliers themselves are identified in Supplementary file 2.
- Data exclusion criteria for calcium imaging data can be found in the Materials and Methods section—simply, heterozygous animals were excluded from analysis.
- Replicate information for the experiments analyzing knockdown efficiency in *lrpprc^gbt0235^*^/^*^gbt0235^* animals can be found in Material and Methods and Figure 3— Source Data 1.
- In these data (Figure 3—Source Data 1), no data was excluded.

**Statistical reporting**

- Statistical analysis methods should be described and justified
- Raw data should be presented in figures whenever informative to do so (typically when N per group is less than 10)
- For each experiment, you should identify the statistical tests used, exact values of N, definitions of center, methods of multiple test correction, and dispersion and precision measures (e.g., mean, median, SD, SEM, confidence intervals; and, for the major substantive results, a measure of effect size (e.g., Pearson's r, Cohen's d)
- Report exact p-values wherever possible alongside the summary statistics and 95% confidence intervals. These should be reported for all key questions and not only when the p-value is less than 0.05.

Please outline where this information can be found within the submission (e.g., sections or figure legends), or explain why this information doesn’t apply to your submission:

- Description and justification for statistical analysis methods can be found in the “Statistical Analysis” section of Materials and Methods and in the figure legends.
- We utilized violin plots and/or presented all data points shown to maximize visualization of data and its distributions.
- Exact values of N, definitions of center, effect size, and statistical tests utilized can be found in the figure legends.
- Summary statistics can be found represented in the plots within the figures along with being found in Supplementary file 2 that contains both the R code and output.
- Exact p-values are reported above each plot within the figures regardless of their value.
- All numeric data needed to calculate mean of knockdown efficiency in tested *lrpprc^mn0235Gt^*^/^*^mn0235Gt^* animals (Figure 3) are documented in Figure 3—Source Data 1.

(For large datasets, or papers with a very large number of statistical tests, you may upload a single table file with tests, Ns, etc., with reference to sections in the manuscript.)

**Group allocation**

- Indicate how samples were allocated into experimental groups (in the case of clinical studies, please specify allocation to treatment method); if randomization was used, please also state if restricted randomization was applied
- Indicate if masking was used during group allocation, data collection and/or data analysis

Please outline where this information can be found within the submission (e.g., sections or figure legends), or explain why this information doesn’t apply to your submission:

- The group allocation for all experiments is described in Materials and Methods section.

**Additional data files (“source data”)**

- We encourage you to upload relevant additional data files, such as numerical data that are represented as a graph in a figure, or as a summary table
- Where provided, these should be in the most useful format, and they can be uploaded as “Source data” files linked to a main figure or table
- Include model definition files including the full list of parameters used
- Include code used for data analysis (e.g., R, MatLab)
- Avoid stating that data files are “available upon request”

Please indicate the figures or tables for which source data files have been provided:

- Figure 3—Source Data 1 is provided as the source data of a part of Figure 3 which is showing knockdown efficiency in *lrpprc^gbt0235^*^/^*^gbt0235^* animals.
- Figure 4—Source Data 1 is provided as the source data summarizing parameters of Ca^2+^ transients in individual animals for Figure 4.
- Figure 4—Source Data 2 is provided as the source ∆F/F­_0_ data for individual calcium transients for Figure 4.
- Figure 4—figure supplement 1—Source Data 1 is provided as the source data summarizing parameters of Ca^2+^ transients in individual cells for Figure 4—figure supplement 1.
- Figure 6—Source Data 1 is provided as the source data for Figure 6A.
- Figure 6—figure supplement 1 is provided as confocal images demonstrating patterns of subcellular localization.
- Supplementary file 3 is provided as the data source of Figure 5B.
- Supplementary file 5 is provided as an Appendix listing the primer sequences.
- All R codes and the outputs are documented and annotated in Supplementary file 6.
